# Digital Peer Support Intervention for Family Caregivers of Individuals With Neuromuscular Disease: Randomized Controlled Trial

**DOI:** 10.2196/86021

**Published:** 2026-07-23

**Authors:** Samantha Mekhuri, Joseph Munn, Francine Buchanan, Nouma Hammash, Munazzah Ambreen, Sara Ahola Kohut, Louise Rose, Reshma Amin

**Affiliations:** 1 Division of Respiratory Medicine, Department of Pediatrics, The Hospital for Sick Children, 555 University Ave, Toronto, ON, M5G 1X8, Canada, 1 647 280-7374; 2 Patient & Family Engagement, The Hospital for Sick Children, Toronto, ON, Canada; 3 Child Health Evaluative Sciences, SickKids Research Institute, Toronto, ON, Canada; 4 Institute of Health Policy, Management and Evaluation, University of Toronto, Toronto, ON, Canada; 5 Spinal Cord Injury Ontario, Toronto, ON, Canada; 6 SickKids Research Institute, The Hospital for Sick Children, Toronto, ON, Canada; 7 Faculty of Nursing, Midwifery & Palliative Care, King’s College London, London, United Kingdom

**Keywords:** neuromuscular diseases, caregivers, peer support, caregiver stress, caregiver burden, long-term home ventilation

## Abstract

**Background:**

Neuromuscular diseases (NMD) affect nerves and muscles, resulting in weakness and often profound disability. Family caregivers of individuals with NMD experience significant psychological burden, stress, and reduced well-being. Digital peer support interventions may help to ameliorate these negative impacts.

**Objective:**

This study evaluated the effect of a 12-week digital peer support intervention compared to usual care on caregiver mastery, competence, stress, burden, anxiety, and depression among family caregivers of individuals with NMD.

**Methods:**

We conducted a parallel-group multicenter randomized controlled superiority trial in Ontario, Canada. Family caregivers of children or adults with NMD were recruited between August 2022 and September 2023 through 7 sites, social media, and national organizations. Participants were randomized 1:1 to a 12-week digital peer support intervention or usual care. The 12-week intervention comprised access to a trained peer mentor, private app-based communication via aTouchAway (Aetonix, Canada), and weekly moderated digital group discussion forums. The primary outcome was caregiver mastery measured using the Pearlin Mastery Scale, adjusted for baseline score. Secondary outcomes included caregiver stress, competence, burden, anxiety, and depression. We calculated adjusted (for baseline score) mean differences using analysis of covariance and generated multivariable linear regression models exploring associations with the intervention and caregiver age, years of caregiving, care recipient medical diagnosis, care recipient ventilation type, adjusting for baseline outcome scores. Intervention fidelity was evaluated through participant engagement metrics.

**Results:**

A total of 100 participants were randomized (n=50 intervention and n=50 control). Participants had a mean age of 46.8 (SD 11.6) years, 70% (n=70) were mothers, with a mean length of caregiving of 11.8 (SD 7.6) years. We found no difference in 12-week Pearlin Mastery Scale scores (adjusted mean difference 0.67, 95% CI −1.7 to 3.1). We also found no difference in any of our secondary outcomes. Mentors and participants sent a mean of 21.3 (SD 33.3) and 17.7 (SD 33.0) messages, respectively. Overall, 62% (n=31) of participants and 92% (n=11) of mentors engaged in at least 1 program element for ≥8 of the 12 weeks.

**Conclusions:**

Our 12-week digital peer support program had no effect on caregiver mastery or other caregiving or psychological outcomes among family caregivers of individuals with NMD. This might be partly due to moderate fidelity and variability in participant engagement. Unlike prior caregiver interventions that incorporated structured psychoeducation or self-management training, this intervention evaluated primarily peer support delivered through a digital platform. This study contributes important evidence regarding the feasibility and limitations of scalable digital peer support programs for caregivers of individuals with NMD. These findings highlight the importance of intervention tailoring, participant matching, and sustained engagement. Future research should evaluate longer-duration and more individualized peer support models targeting caregivers earlier in the caregiving trajectory to improve intervention fidelity and ultimately caregiver well-being.

## Introduction

Family caregivers are essential for the care of individuals with neuromuscular disease (NMD) in the home, particularly those using home mechanical ventilation (HMV) [1]. Caregivers of individuals with NMD frequently provide complex long-term physical, emotional, and medical support, including respiratory care and coordination of multidisciplinary services [2,3]. Consequently, family caregiving is associated with significant burden, social isolation, and negatively affects the caregiver’s physical and mental health [2,3]. Caregivers have reported unmet psychosocial and support needs and value peer connection as a means of alleviating these challenges [3-5].

Mastery and caregiver competence are important constructs that may improve the ability to cope with caregiving-related stressors [6-8]. Mastery refers to the perceived sense of control over life situations [6-8]. Caregiver competence is the self-appraisal of caregiving adequacy and performance [6-8]. The Stress Process Model describes caregiver stress as arising from four interrelated domains: caregiving background and stress context, stressors, stress mediators, and stress outcomes or manifestations [6-8]. Within this framework, social support may act as a stress mediator that mitigates the psychological impact of caregiving demands, as greater social support is associated with higher levels of mastery and caregiver competence [9,10]. Therefore, mastery and caregiver competence may represent modifiable protective factors against the psychological stressors associated with caregiving [8-10].

Peer support includes emotional, informational, and affirmational support by individuals with lived experience of a similar health problem [11-13]. Through shared experiences, validation, normalization of caregiving challenges, and exchange of caregiving strategies, peer support may enhance caregivers’ perceived sense of control and self-efficacy, thereby strengthening mastery and competence [4,9,10,14]. Previous studies have demonstrated that peer support may improve health-related quality of life, self-efficacy, and empowerment while reducing stress across various patient and caregiver populations [4,15,16]. Digital peer support interventions may further facilitate the development of mastery through shared learning, emotional support, and increased social connectedness [14]. Given caregiving commitments, in-person peer support is challenging due to geographic limitations and time constraints. Hence, digitally delivered peer support is ideal for caregivers of individuals with NMD who have high care needs [5,17]. However, evidence supporting the effectiveness of digital peer support interventions for caregivers of individuals with NMD remains limited, and few programs have been formally evaluated [4,18-20].

We previously designed and beta-tested a digital peer support program for caregivers of individuals using HMV with an asynchronous interactive chat forum hosted on a bespoke website. These interactions underscored the complexities of caring for an individual requiring HMV [18]. Building on this prior work, the primary objective of this study was to evaluate the effect of a digital peer support intervention for family caregivers of children and adults with NMD on caregiver mastery compared to usual care. Secondary objectives were to determine the effect on caregiver competence, stress, burden, anxiety, and depression.

## Methods

### Trial Design

We conducted a parallel-group, multicenter, randomized controlled superiority trial. The study was prospectively registered on ClinicalTrials.gov (NCT05070624) on September 27, 2021, prior to enrollment of the first participant on September 2, 2022.

This study was conducted and reported in accordance with the CONSORT (Consolidated Standards of Reporting Trials) 2025 statement (Checklist 1), the CONSORT E-HEALTH checklist (Checklist 2), and the TIDieR (Template for Intervention Description and Replication) checklist (Checklist 3) [21-23].

### Changes to Trial Protocol

Three protocol amendments were made following trial registration and ethics approval. The primary and secondary outcomes remained unchanged throughout the study.

#### Amendment 1 (August 23, 2022)

Recruitment expanded from participating clinical sites to caregivers across Canada to improve sample diversity and feasibility. Recruitment through social media and community organizations was added, along with an electronic expression of interest form and revised recruitment materials. Follow-up was extended from 12 to 24 weeks to assess the effects of the intervention after completion of the intervention at 12 weeks.

#### Amendment 2 (March 17, 2023)

The Center for Epidemiologic Studies Depression Scale was removed as an eligibility criterion to reduce participant burden because psychological outcomes were already assessed using the Depression Anxiety Stress Scales-21 (DASS-21).

#### Amendment 3 (June 23, 2023)

Eligibility criteria were broadened from caregivers of individuals with NMD using HMV to caregivers of all individuals with NMD. References to the COVID-19 pandemic were removed to improve applicability in the postpandemic context, and study materials were revised accordingly.

All amendments received ethics approval before implementation and were updated in the trial registration record prior to completion of data analysis.

### Patient and Public Involvement

Family caregivers of individuals with neuromuscular disease contributed to the development and refinement of the digital peer support intervention through prior beta-testing and feedback on intervention content, delivery format, and discussion topics. Peer mentors with lived caregiving experience also informed the design of the mentor training procedures and weekly discussion sessions [18]. Patients and the public were not involved in recruitment, data analysis, or interpretation of study findings.

### Trial Setting and Recruitment

We recruited participants and mentors from 7 sites across Ontario, Canada, through self-referral via social media and national charities and organizations. Participants were recruited from August 2022 to September 2023 (see Multimedia Appendix 1 for study sites, relevant charities and organizations, and trial design). Interested individuals completed an expression of interest form sent via email to the study coordinating center to determine eligibility and were subsequently recruited by research coordinators.

### Eligibility Criteria

Inclusion criteria for study participants were (1) family caregiver of an adult or child with NMD living in Canada, (2) speaks and reads English, (3) access to the internet and a computer or tablet, and (4) consents to participation. Inclusion criteria for peer mentors were the same as those for participants, with the additional requirement of completing our digital peer support training. No exclusion criteria were applied.

### Ethical Considerations

This trial received research ethics approval through Clinical Trials Ontario (CTO-3590). Participants and mentors provided written electronic informed consent using the REDCap (Research Electronic Data Capture; Vanderbilt University) database in accordance with the Declaration of Helsinki [24].

Participant privacy and confidentiality were protected through the use of unique study identification numbers and deidentified data analysis. Study data were securely managed using REDCap hosted at The Hospital for Sick Children, and TouchAway (Aetonix, Ottawa, Canada) data were stored on secure Canadian cloud servers in accordance with Ontario’s Personal Health Information Protection Act. Access to identifiable data was restricted to authorized study personnel. All reported findings are presented in aggregate form to protect participant confidentiality.

Upon study completion, participants were provided a CAD $30 (US $21.11; exchange rate CAD $1=US $0.70, converted on July 6, 2026) online gift card.

No participant-identifying information is included in this manuscript or supplementary materials.

### Randomization and Blinding

Research coordinators enrolled eligible participants, and participants were randomized through the REDCap randomization module. Participants were randomized in a 1:1 ratio using a computer-generated randomization schedule with random permuted block sizes of 2 or 4 generated by an independent statistician. Allocation concealment was maintained through the REDCap randomization module, which was inaccessible to study personnel until assignment. Due to the nature of the intervention, participants, peer mentors, and study personnel were not blinded to the group allocation. Outcome assessors were also not blinded due to resource limitations.

### Intervention and Comparator

#### Digital Peer-Support Program

The intervention was informed by prior pilot and qualitative work suggesting that digitally delivered peer support from individuals with shared caregiving experience may provide emotional, informational, and social support for caregivers of individuals with NMD [18,25]. A 12-week intervention period was selected to allow sufficient time for peer relationship development, support, and sustained participant engagement in the program [18,25,26].

We adapted our previously beta-tested peer support program for delivery on the aTouchAway app. This included peer mentor training, private chat between peers, public discussion forums, and weekly moderated group chats. The 12-week digital peer-support intervention comprised (1) private communication (SMS text messaging [asynchronous], voice calls, or video calls [synchronous]) with a peer mentor via the aTouchAway app, (2) asynchronous discussion forum (group chat) via app messaging, and (3) weekly synchronous group discussion sessions hosted via Zoom.

Weekly discussion sessions were 1 hour in duration, with a range of topics relevant to family caregivers discussed. This included caregiving, health system navigation, stress management, accessibility for individuals with disabilities, transitioning to adult care, disability and reproductive justice, and mental health. A content expert, that is, someone with specialized knowledge in a specific topic, was present at each session, giving either a formal presentation and/or guiding discussion. These sessions were digitally recorded and shared with all intervention participants if they were unable to attend in real time.

Participants were matched with a peer mentor based on two factors: (1) adult or child care recipient and (2) use of HMV. Peer mentors were family caregivers of individuals with NMD with prior lived caregiving experience who completed standardized mentor training before participation. Mentors supported between 1 and 7 participants, averaging 3 to 4 participants per mentor. Participants randomized to the intervention arm were onboarded to the aTouchAway app by a research coordinator, with instructions provided over the telephone on how to download and use the app while addressing any concerns.

#### Peer Mentor Training

We previously adapted and tested a digital peer mentor training program from St Jude Research Hospital (Memphis, TN, USA) program for caregivers of children with cancer [25], with subsequent refinement and use in an ongoing United Kingdom trial of caregiver peer support [26]. Twelve peer mentors underwent four 1-hour training sessions (led by FB, NH, and SM) held virtually using Zoom. Training focused on mentorship skills building, case scenarios, boundary setting, when to seek help, and ensuring personal well-being as a mentor. A research coordinator checked in with mentors every 2 weeks to address any questions or concerns and to facilitate assistance with participant connection as needed.

Participants and mentors were instructed that private communication should occur a minimum of once weekly and asynchronous group chat a minimum of twice weekly. Mentors were instructed to contact the study team, which included social work and psychiatry members, if concerned about a participant’s well-being. Apart from mentor matching based on caregiving context, intervention components and content were standardized across participants and were not otherwise personalized. Nonconfidential and nonproprietary peer mentor training materials, onboarding information, and weekly discussion topic guides are available from the corresponding author upon request.

#### Control Arm

Participants randomized to the control arm continued to receive usual care, consisting of any health care, community, or psychosocial support services accessed independently outside of the study. The research team did not provide any additional or alternative support. Upon trial completion, these participants were provided access to the peer support discussion session recordings and resources but had no access to real-time peer mentoring or live group discussions.

### Study Outcomes

Our primary study outcome was caregiver mastery measured using the Pearlin Mastery Scale (PMS) at 12 weeks and adjusted for baseline score. The PMS is a valid and reliable 7-item scale [27] with scores ranging from 7 to 49; higher scores indicate higher levels of mastery [28]. The PMS has been used in previous studies of caregivers of people with various conditions, including respiratory failure and cancer [29-32], and studies of web-based interventions [17]. We also measured PMS at 24 weeks.

Secondary outcomes were measured at baseline, 12 weeks, and 24 weeks and included (1) caregiver stress and competence (Caregiving Stress Scale [CSS]), (2) caregiver burden (Zarit Burden Interview [ZBI]), and (3) depression, anxiety, and stress (Depression and Anxiety Stress Scale-21 [DASS-21]).

The CSS is a valid and reliable tool [33,34] comprising 15 independent subscales (score range 1‐4); higher scores reflect better outcomes [7]. This scale is used in caregiving research to assess self-appraisal of efficacy in caregiving [35-37]. The ZBI is a valid and reliable tool widely used to evaluate caregiver burden [38-40]. It has 22 items with total scores ranging from 0 to 88; higher scores reflect higher levels of caregiver burden [41]. The DASS-21 is a valid and reliable 21-item tool [42,43] with subscale scores ranging from 0 to 42; higher scores indicate higher levels of depression, anxiety, or stress [44]. Participants completed the Caregiving Assistance Scale at baseline to assess caregiving contextual factors [45].

### Intervention Fidelity

Via the aTouchAway app, we collected data on text message, phone, and video call volume sent by mentors and participants and monitored participation in weekly discussion sessions. We established the following criteria to determine fidelity [25]:

1. ≥60% of participants and mentors engage in any program element (ie, private chat and discussion sessions) for ≥8 per 12 weeks.
2. ≥50% of participants and mentors join the weekly discussion session for ≥8 of the 12 weeks.
3. ≥50% of participants post on the asynchronous discussion forum for ≥8 of the 12 weeks.
4. ≥50% of participants contact a mentor during the 12-week program for ≥8 of the 12 weeks.
5. Attrition: ≤30% of participants withdraw from the study before completion of postintervention questionnaires.

### Harms

Given the nature of the intervention, risks to participants were considered minimal. Potential harms included emotional distress during peer support discussions, including disclosures of caregiver challenges. Peer mentors were trained to recognize participant distress and escalate concerns to the study team, which included social workers and psychiatry professionals.

### Data Collection

All study data were collected using case report forms developed in the REDCap database by trained research personnel. Participants completed study measures either independently via this database accessed through an email link with up to 3 reminder prompts or with assistance from a research coordinator via telephone, according to their preference.

### Sample Size

Basing our assumptions on a previous study of a web-based intervention for family caregivers [17], we assumed a minimum clinically important difference in the PMS of 2.95 points, an SD of 4.5, with 90% power and α of 5%. We required a sample size of 50 study participants in each group (n=100 participants total). Therefore, the study was appropriately powered to detect a clinically meaningful difference in the PMS. We completed a post hoc power analysis to identify the minimum detectable difference for the primary outcome. As the initial sample size calculation was performed using a version of the PMS with scores ranging from 7 to 28 as opposed to 7 to 49, the minimum clinically important difference for this study was adjusted to 5.16 with an SD of 7.87 for the post hoc analysis.

### Statistical Methods

#### Data Analysis

All analyses were completed using the intention-to-treat principle.

We summarized baseline characteristics and intervention fidelity using mean (SD) and n (%). We used analysis of covariance to calculate adjusted mean differences and 95% CIs for our primary and secondary outcomes, adjusting for the baseline score. We used the Holm adjustment to control for multiple testing [46].

We conducted a priori planned exploratory analyses using multivariable linear regression models exploring associations of caregiver age, years of caregiving, care recipient medical diagnosis, ventilation type, and intervention arm while controlling for baseline outcome score.

All statistical analyses were conducted using R software (version 4.4.1) [47] by an independent statistician.

#### Missing Data

Participant-level and item-level missing data were captured. To assess whether data were missing completely at random (MCAR), Little MCAR test was performed. Missing data were imputed using multiple or single imputation depending on the number of missing observations, with single imputation used if ≤1% of data were missing.

## Results

### Study Overview

#### Participant Enrollment

**Figure 1.**
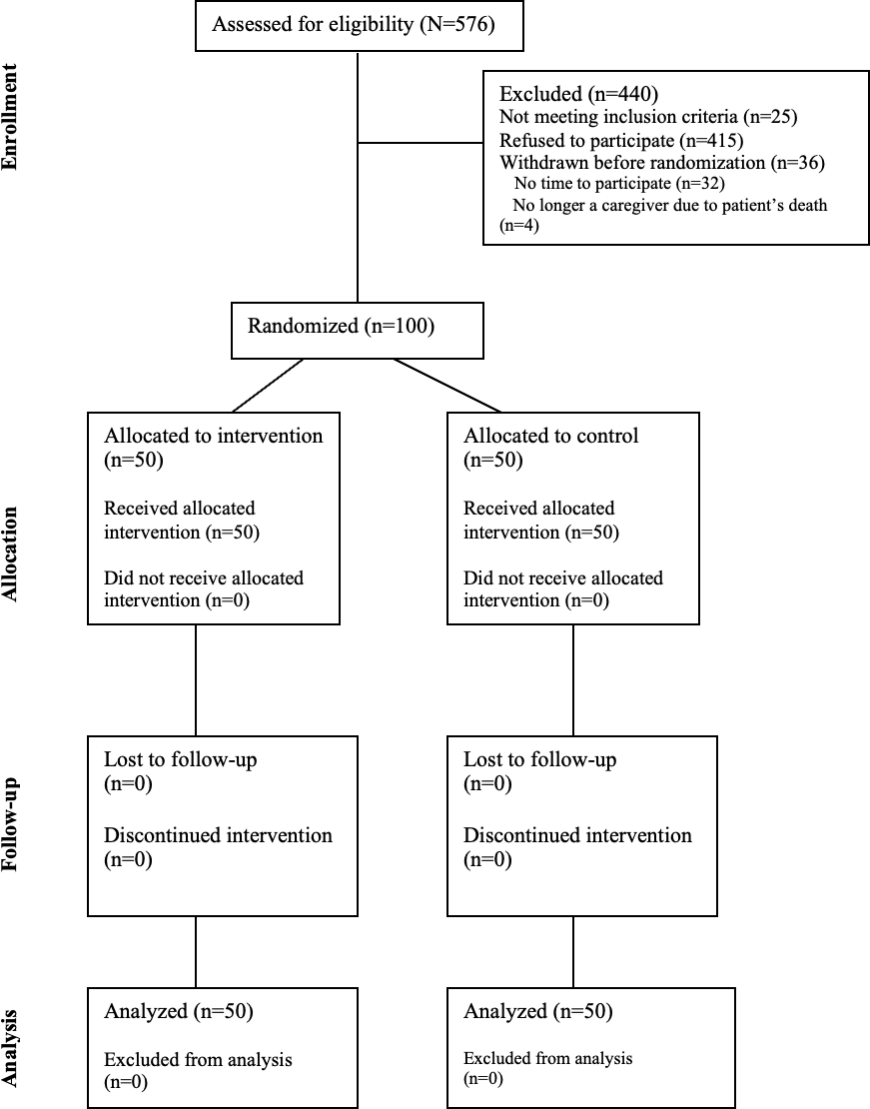
CONSORT (Consolidated Standards of Reporting Trials) flow diagram of participant recruitment, enrollment, randomization, allocation, follow-up, and analysis for the 12-week peer support intervention. The diagram shows the number of participants assessed for eligibility, excluded (with reasons), randomized to the intervention and control groups, lost to follow-up, discontinued from the intervention, and included in the final analysis.

We screened 576 family caregivers referred by our recruiting centers or through self-referral. Of these, 25 (4%) did not meet inclusion criteria, 415 (72%) declined to participate, and 36 (6%) caregivers initially gave written or verbal consent but withdrew before randomization. We randomized 100 participants (50 to each arm), with 99 completing the study and providing the primary outcome (ie, 1% attrition) (Figure 1). Participants were recruited between August 2022 and September 2023, and study follow-up was completed by March 2024.

#### Participant Characteristics

Intervention arm participants had a mean age of 45.6 (SD 12.5) years, with 76% (n=38) identifying as a woman. Care recipients of intervention participants had a mean age of 21.5 (SD 20.5) years; children had a mean age of 12.0 (SD 6.5) years, and adults had a mean age of 55.5 (SD 16.5) years. The most common diagnoses were muscular dystrophy (40%) and spinal muscular atrophy (SMA; 22%).

Control arm participants had a mean age of 48.1 (SD 10.7) years, with 86% (n=43) identifying as a woman. Care recipients of control participants had a mean age of 22.2 (SD 19.5) years; children had a mean age of 14.0 (SD 6.4) years, and adult care recipients had a mean age of 55.5 (SD 19.8) years. The most common diagnoses were muscular dystrophy (44%) and SMA (12%) (Table 1).

**Table 1.** Participant baseline characteristics stratified by assignment to the intervention and control groups, with continuous variables presented as means (SD) and categorical variables presented as n (%).

Characteristics	Control (n=50)	Intervention (n=50)
Caregiver characteristics		
Age (y), mean (SD)	48.10 (10.69)	45.58 (12.48)
Gender, n (%)		
Woman	43 (86)	38 (76)
Man	7 (14)	12 (24)
Relationship to care recipient, n (%)		
Mother	37 (74)	33 (66)
Father	3 (6)	8 (16)
Other	10 (20)	9 (18)
Length of caregiving (y), mean (SD)	13.0 (7.9)	10.7 (7.2)
Ethnicity, n (%)		
White	21 (42)	28 (56)
South Asian	10 (20)	11 (22)
Asian	4 (8)	5 (10)
Arab	5 (10)	2 (4)
Latin American	4 (8)	0 (0)
Black	3 (6)	1 (2)
Prefer not to say	2 (4)	2 (4)
First Nations	0 (0)	1 (2)
Mixed race	1 (2)	0 (0)
Education level, n (%)		
Received university or college degree or diploma	34 (68)	38 (76)
Some postsecondary education (university or college)	6 (12)	7 (14)
Completed secondary school	5 (10)	4 (8)
Did not complete secondary school	5 (10)	1 (2)
Employment, n (%)		
Full-time	21 (42)	21 (42)
Other	29 (48)	29 (58)
Gross household income (US $), n (%)		
Less than 50,000	7 (14)	8 (16)
50,000-79,999	4 (8)	2 (4)
80,000-99,999	13 (26)	10 (20)
100,000 or more	12 (24)	21 (42)
Prefer not to answer	14 (28.0)	9 (18.0)
Care recipient characteristics		
Age (y), mean (SD)	22.21 (19.50)	21.55 (20.47)
Adult care recipients, age (y), mean (SD)	55.10 (19.77)	55.45 (16.48)
Pediatric care recipients, age (y), mean (SD)	13.99 (6.40)	11.98 (6.49)
Diagnosis, n (%)		
Muscular dystrophy	22 (44)	20 (40)
Spinal muscular atrophy	6 (12)	11 (22)
Myopathy	5 (10)	5 (10)
Amyotrophic lateral sclerosis	4 (8)	3 (6)
Other	13 (26)	11 (22)
HMV^a^, n (%)		
Noninvasive	32 (64)	28 (56)
None	12 (24)	17 (34)
Invasive	6 (12)	5 (10)

a HMV: home mechanical ventilation.

#### Adverse Events

No serious adverse events or harms related to study participation were reported during the trial.

#### Missing Data

Less than 1% of participant-level data were missing. One participant was lost to follow-up at the 12-week time point and did not complete the 12-week PMS, ZBI, DASS-21, and CSS questionnaires, and 2 individuals were lost to follow-up at the 24-week time point and did not complete the 24-week PMS, ZBI, DASS-21, and CSS questionnaires. Little MCAR test indicated that the data were MCAR (*P*=.93). No other data were missing. As a small amount of data were missing (<1%), single imputation using predictive mean matching was performed.

### Primary Outcome Analysis

Mean (SD) PMS scores at 12 weeks were 29.8 (9.3) for the intervention group and 29.4 (8.7) for the control group, with an adjusted mean difference of 0.67 (95% CI −1.75 to 3.10; Table 2).

**Table 2.** Primary and secondary outcome measures, including the Pearlin Mastery Scale, Zarit Burden Interview, Depression Anxiety Stress Scales-21, and Caregiver Stress Scale caregiver competence, stratified into intervention, control, and overall groups at baseline, 12 weeks, and 24 weeks.^a^

Outcome measure	Control (n=50), mean (SD)	Intervention (n=50), mean (SD)	Overall (N=100), mean (SD)
Pearlin Mastery Scale			
Baseline	31.20 (9.19)	30.92 (8.01)	31.06 (8.58)
12 weeks	29.36 (8.71)	30.52 (9.39)	29.94 (9.03)
24 weeks	28.78 (7.71)	28.50 (7.79)	28.64 (7.71)
Zarit Burden Interview			
Baseline	31.26 (15.02)	29.98 (15.16)	30.62 (15.03)
12 weeks	33.14 (15.02)	32.84 (13.03)	32.99 (13.99)
24 weeks	34.24 (16.89)	35.42 (13.64)	34.83 (15.28)
DASS-21^b^ depression			
Baseline	7.44 (7.13)	9.16 (8.06)	8.30 (7.62)
12 weeks	6.64 (5.50)	9.16 (8.32)	7.90 (7.13)
24 weeks	7.88 (7.68)	8.64 (8.6)	8.26 (8.12)
DASS-21 anxiety			
Baseline	5.56 (6.11)	5.88 (6.68)	5.72 (6.37)
12 weeks	5.08 (5.38)	5.68 (7.54)	5.38 (6.52)
24 weeks	5.64 (6.83)	5.16 (6.36)	5.4 (6.57)
DASS-21 stress			
Baseline	10.84 (6.79)	12.12 (8.13)	11.48 (7.48)
12 weeks	10.88 (6.71)	11.28 (8.02)	11.08 (7.36)
24 weeks	12.2 (7.73)	12.08 (8.81)	12.14 (8.25)
CSS^c^ caregiving competence			
Baseline	13.94 (2.03)	12.90 (2.48)	13.42 (2.31)
12 weeks	13.60 (2.16)	12.96 (2.36)	13.28 (2.27)
24 weeks	13.70 (2.35)	13.44 (2.71)	13.57 (2.53)

a All results are presented as unadjusted mean (SD).

b DASS-21: Depression and Anxiety Stress Scale-21.

c CSS: Caregiver Stress Scale.

### Secondary Outcome Analysis

We found no difference between intervention and control group scores for PMS measured at 24 weeks or the ZBI, DASS-21, or CSS measured at 12 and 24 weeks (Table 3).

**Table 3.** Results of analysis of covariance comparing intervention and control groups at 12 and 24 weeks for primary and secondary outcomes, including Pearlin Mastery Scale, Zarit Burden Interview, Depression Anxiety Stress Scale-21, and Caregiver Stress Scale caregiver competence.^a^

Variables	12 weeks			24 weeks		
	aMD^b^ (95% CI)	*P* value	Adjusted *P* value	aMD (95% CI)	*P* value	Adjusted *P* value
Pearlin Mastery Scale	0.67 (−1.75 to 3.10)	.59	>.99	-0.40 (−2.83 to 2.03)	.75	>.99
Zarit Burden Interview	1.78 (−1.67 to 5.22)	.31	>.99	0.93 (−3.46 to 5.32)	.68	>.99
DASS-21^c^						
Depression	1.13 (−0.69 to 2.96)	.23	>.99	−0.39 (−2.75 to 1.96)	.74	>.99
Anxiety	0.45 (−1.42 to 2.31)	.64	>.99	−1.18 (−3.45 to 1.09)	.31	>.99
Stress	−0.49 (−2.56 to 1.58)	.64	>.99	−0.92 (−3.46 to 1.61)	.48	>.99
CSS^d^ caregiving competence	0 (−0.71 to 0.70)	.99	>.99	−0.03 (-0.98 to 0.92)	.96	>.99

a Results are presented as adjusted mean differences with 95% CIs. Unadjusted *P* values and *P* values adjusted using the Holm method are included.

b aMD: adjusted mean difference.

c DASS-21: Depression and Anxiety Stress Scale-21.

d CSS: Caregiving Stress Scale.

### Exploratory Analysis

Controlling for baseline PMS score, we found no association between the 12-week PMS score and caregiver age, years of caregiving, medical diagnosis, ventilation type, and randomization arm (Table 4).

Exploratory analysis of secondary outcomes (Multimedia Appendix 2) found the following associations, controlling for baseline scores: fewer caregiving years (estimate −0.23, 95% CI −0.45 to −0.01) was associated with a lower 24-week PMS score, indicating lower mastery; older caregiver age (estimate 0.11, 95% CI 0.1 to 0.22) and care recipient SMA diagnosis (reference: amyotrophic lateral sclerosis [ALS]; estimate 5.21, 95% CI 0.11 to 10.31) were associated with higher 12-week and 24-week (SMA compared to ALS; estimate 7.07, 95% CI 1.04 to 13.09) DASS anxiety scores, indicating more psychological morbidity. Conversely, being a man was associated with lower 24-week DASS anxiety scores (estimate −3.01, 95% CI −5.98 to −0.03) and 24-week DASS stress scores (estimate −4.82, 95% CI −8.15 to −1.49), indicating less psychological morbidity. Caregivers of those with a myopathy diagnosis had lower 24-week CSS caregiver competency scores (reference: ALS; estimate −2.68, 95% CI −5.32 to −0.05), indicating less caregiver stress.

**Table 4.** Linear regression models exploring variables associated with the Pearlin Mastery Scale (PMS) score measured at 12 and 24 weeks, controlling for baseline PMS scores.

Predictors	Pearlin 12 weeks		Pearlin 24 weeks	
	Estimate (95% CI)	*P* value	Estimate (95% CI)	*P* value
Allocation (reference: control)				
Intervention	0.54 (−1.99 to 3.07)	.67	−0.29 (−2.87 to 2.30)	.83
Pearlin baseline	0.77 (0.62 to 0.92)	<.001^a^	0.56 (0.41 to 0.71)	<.001^a^
Caregiving years	−0.03 (−0.24 to 0.18)	.77	−0.23 (−0.45 to −0.01)	.04^a^
Caregiver age	-0.14 (−0.28 to 0.00)	0.06	0.14 (−0.01 to 0.28)	.06
Caregiver gender (reference: woman)				
Man	0.38 (−3.00 to 3.76)	.82	0.92 (−2.53 to 4.36)	.60
Diagnosis (reference: ALS)^b^				
Muscular dystrophy	−1.44 (−7.97 to 5.09)	.66	5.41 (−1.25 to 12.07)	.11
Myopathy	−4.22 (−11.27 to 2.82)	.24	4.3 (−2.88 to 11.48)	.24
Other	0.06 (−6.64 to 6.77)	.99	6.01 (−0.82 to 12.85)	.08
Spinal muscular atrophy	−4.34 (−11.20 to 2.52)	.21	4.48 (−2.51 to 11.48)	.21
HMV^c^ (reference: invasive**)**				
None	1.13 (−3.23 to 5.49)	.61	1.93 (−2.51 to 6.38)	.39
Noninvasive	0.93 (−3.22 to 5.09)	.66	1.05 (−3.19 to 5.28)	.63

a Statistically significant *P* values.

b ALS: amyotrophic lateral sclerosis.

c HMV: home mechanical ventilation.

### Program Fidelity

Thirty-one (62%) intervention participants and 11 (92%) mentors engaged with at least 1 program component for 8 of the 12 weeks. Among the specific elements, participants and mentors interacted most frequently through app-based messaging. Twenty-one (42%) participants contacted their mentor during 8 of the 12 weeks. Peer mentors sent a mean of 21.3 (SD 33.3) messages per participant they mentored, while participants sent a mean of 17.7 (SD 33.0) messages to their respective mentor (Table 5).

Fifteen (30%) intervention participants and 2 (17%) mentors attended the weekly discussion sessions for 8 of 12 weeks. Only 4 (8%) participants posted on the asynchronous discussion forum for 8 of 12 weeks. One (2%) participant withdrew after completing the 12-week program but before completing postintervention questionnaires.

**Table 5.** Patient program fidelity measures indicating the frequency with which individuals included in the intervention arm of the study made use of the various components of the intervention, presented as mean (SD) and median (IQR).

Variables	Overall (n=50)
Texts sent by participant	
Mean (SD)	17.72 (33.03)
Median (IQR)	4.0 (2.0-14.8)
Texts sent to participant	
Mean (SD)	21.28 (33.26)
Median (IQR)	8.0 (4.0-18.8)
Video calls to participant	
Mean (SD)	0.12 (0.59)
Median (IQR)	0.0 (0.0-0.0)
Video calls from participant	
Mean (SD)	0.22 (0.68)
Median (IQR)	0.0 (0.0-0.0)
Voice calls to participant	
Mean (SD)	0.06 (0.31)
Median (IQR)	0.0 (0.0-0.0)
Voice calls from participant	
Mean (SD)	0.18 (0.52)
Median (IQR)	0.0 (0.0-0.0)

## Discussion

### Summary and Interpretation of Findings

In this randomized controlled trial of a 12-week digital peer-support intervention for family caregivers of children and adults with NMD, we found no difference between the intervention and control arms for our primary outcome of caregiver mastery measured at 12 weeks and adjusted for baseline score. Similarly, there were no differences in our secondary outcomes, including caregiver stress, competence, burden, anxiety, and depression. Our exploratory analyses found that fewer caregiving years were associated with less mastery, while older caregiver age and caregiving for an individual with SMA were associated with worse psychological outcomes. These findings suggest that digitally delivered peer support alone may be insufficient to improve psychosocial outcomes in a heterogeneous population of caregivers with established caregiving experience.

Our study did not observe an effect on caregiver mastery, an outcome that has been infrequently examined in previous trials of digital peer support interventions [48,49]. Caregiver mastery is defined as a positive belief in one’s own ability to manage the challenges of caregiving [28]. Mastery is derived from a sense of ability to handle caregiving demands and perceived self-efficacy and is protective against stress and the negative mental health sequelae of caregiver burden [17,28,31]. We therefore chose caregiver mastery as our primary outcome measure, as this best reflected the intended benefits of our virtual peer support program. However, other previous studies on digital peer support for caregivers have focused on outcomes such as caregiving competence, burden, stress, depression, and anxiety [50-56]. Among studies that did measure caregiver mastery, the interventions were mainly structured educational programs or personalized coaching [48,49]. For example, mastery, measured as a secondary outcome, was significantly improved using an 8-week self-management program for caregivers of individuals with dementia, combining face-to-face coaching by trained psychologists with online forums for peer interaction [52]. In a trial of a web-based intervention for stroke patients and their caregivers that included both peer and professional support via online chat sessions, no direct effect on mastery (secondary outcome) was found; however, reductions in caregiver depression were significantly associated with increased mastery [56]. These studies suggest that caregiver mastery may be more responsive to interventions incorporating self-management or psychoeducational strategies rather than to peer support alone.

In our exploratory analyses, we found that a shorter caregiving duration was associated with lower mastery. This may reflect the challenges faced by newer caregivers who are still adapting to their roles and lack established coping strategies [57]. Older caregiver age and an SMA diagnosis were associated with higher anxiety. Anxiety has been linked to increased health concerns and care demands, physical and mental fatigue, social isolation, financial strain, and difficulty engaging with digital platforms [58-65]. Men caregivers reported lower psychological morbidity compared to women caregivers, consistent with prior research [66-68]. Myopathy diagnosis correlated with lower caregiver competence, likely reflecting the condition’s complexity and limited support [69]. It is also important to note that the CIs for these findings were quite wide, limiting our confidence in the precision of the estimates and their effect on the outcomes of interest.

Several intervention-related and participant-related factors may explain the absence of measurable intervention effects on caregiver mastery. First, the 12-week intervention duration may be too short to produce an effect on mastery. Second, inclusion of caregivers for children and adults with any form of NMD introduced sample heterogeneity, potentially limiting the ability to foster meaningful peer connections [70]. Third, the digital format may have reduced interaction quality and, therefore, homophily [71,72]. Fourth, some peer mentors were active caregivers themselves while responsible for supporting multiple participants, which may have diluted their capacity to provide consistent or individualized peer support. Fifth, baseline mastery scores were in the midrange, suggesting participants may already possess a strong sense of mastery, limiting the potential for improvement. Sixth, although it is possible that the lack of effect observed in this trial was due to insufficient power, this is unlikely, as post hoc analyses suggested the study was adequately powered to detect a clinically meaningful difference in caregiver mastery.

Intervention fidelity and participant engagement may also have contributed to the absence of measurable effects. Engagement with several intervention components was low, particularly the asynchronous discussion forum and weekly group sessions. Our qualitative feedback suggested barriers such as time constraints and difficulty feeling connected to mentors due to differences in caregiving characteristics [73], indicating that more tailored matching or flexible delivery approaches warrant further exploration. While digital interventions help to address barriers such as social isolation and geographical distance, they may not consistently support meaningful connections [49,74].

### Strengths and Limitations

A key strength of this study is its rigorous randomized controlled design, which minimizes bias through randomization and allocation concealment. However, as participants and outcome assessors were not blinded, there is potential for performance and detection bias, particularly given the use of self-reported psychosocial outcomes. As no significant between-group differences were observed, any such bias was unlikely to have substantially affected the findings. Additionally, a large proportion of eligible caregivers declined participation, introducing potential selection bias, which may limit the generalizability of our findings. It is possible that those who agreed to participate were more confident or capable in their caregiving roles and thus more willing to engage in peer support. Those who declined may have been the individuals who would have benefited most from the intervention. Allowing participants to choose their peer mentor may have enhanced relational depth and tailored support. However, this was not feasible given our small pool of peer mentors. Most participants were existing caregivers with moderate to high mastery and low distress, suggesting our inclusion criteria could have included measurement of baseline mastery to identify those most in need of the intervention. Although the peer mentor training program was adapted from a previously established caregiver support model and refined through pilot testing with caregivers of individuals with NMD, the original framework was developed for caregivers of children with cancer. While core mentorship principles such as communication skills, boundary setting, and emotional support are transferable, the training may not have fully addressed the unique challenges associated with NMD caregiving, such as progressive functional decline, long-term ventilation support, and complex care coordination [75,76]. For the exploratory component of the analysis, no corrections were made for multiple comparisons or false discovery. This limits the confidence that can be placed in the results of this portion of the study, and future work is necessary to confirm these findings. Lastly, this study did not directly measure stress mediators that social support is theorized to affect by the Stress Process Model, specifically social support, self-efficacy, and competence. This limits our ability to understand why the intervention was not effective within this population. Although the study did capture depression, anxiety, stress, burden, and caregiver sense of mastery, more direct measures would likely have improved our understanding of the direct effects of the intervention.

### Conclusion

This randomized controlled trial suggests that digitally delivered peer support alone may be insufficient to improve psychosocial outcomes among caregivers of individuals with NMD, particularly within a heterogeneous population of experienced caregivers. While several factors, including the short intervention duration, potential ceiling effects, lack of homophily, and variability in caregiver engagement, may have contributed to the absence of measurable impact, these explanations remain speculative. Future research should consider focusing on caregivers who are newer to the caregiving role and evaluating longer-duration, individually tailored peer support interventions integrating psychoeducational or skills-based components to better understand their potential to improve caregiver well-being. These findings highlight the challenges of designing scalable digital support interventions that foster meaningful interpersonal connection while addressing the diverse needs of family caregivers.

## Supplementary material

10.2196/86021 Multimedia Appendix 1 Recruitment overview.

10.2196/86021 Multimedia Appendix 2 Exploratory analyses.

10.2196/86021 Checklist 1 CONSORT checklist.

10.2196/86021 Checklist 2 CONSORT-eHEALTH checklist (V 1.6.1).

10.2196/86021 Checklist 3 TIDieR checklist.

## Abbreviations:

ALS: amyotrophic lateral sclerosis
CONSORT: Consolidated Standards of Reporting Trials
CSS: Caregiver Stress Scale
DASS-21: Depression, Anxiety, and Stress Scale-21
HMV: home mechanical ventilation
MCAR: missing completely at random
NMD: neuromuscular disease
PMS: Pearlin Mastery Scale
SMA: spinal muscular atrophy
TIDieR: Template for Intervention Description and Replication
ZBI: Zarit Burden Interview
