# Supplementary material for: Digital Peer Support Intervention for Family Caregivers of Individuals With Neuromuscular Disease: Randomized Controlled Trial
Source: J Med Internet Res. 2026 Jul 23;28:e86021. doi: 10.2196/86021 (PMC13394866; doi:10.2196/86021)
Supplement: Multimedia Appendix 1 [file jmir-v28-e86021-s001.docx]

**Appendix 3:** Recruitment Overview

We recruited study participants and peer mentors through clinician (respirology and neurology) referrals from seven centers:

- The Hospital for Sick Children
- McMaster Children’s Hospital
- Children’s Hospital of Eastern Ontario
- Sunnybrook Hospital
- London Health Sciences
- The Ottawa Hospital
- West Park Healthcare Centre

We also recruited participants through social media, and relevant charities and organizations across Canada:

- Muscular Dystrophy Canada
- Amyotrophic Lateral Sclerosis Society
- Cure Spinal Muscular Atrophy (SMA) Canada
- Multiple Sclerosis Society
- Canadian Organization for Rare Disease
- Canadian Neuromuscular Disease Network
