# Supplementary material for: Digital Peer Support Intervention for Family Caregivers of Individuals With Neuromuscular Disease: Randomized Controlled Trial
Source: J Med Internet Res. 2026 Jul 23;28:e86021. doi: 10.2196/86021 (PMC13394866; doi:10.2196/86021)
Supplement: Multimedia Appendix 2 [file jmir-v28-e86021-s002.docx]

| **Table 1.** Linear regression models investigating additional predictors of the Depression Anxiety Stress Scale anxiety measure at 12 and 24 weeks controlling for baseline scores. | | | | | | | |
| --- | --- | --- | --- | --- | --- | --- | --- |
|  |  | **DASS Anxiety 12 Weeks** | | | **DASS Anxiety 24 Weeks** | | |
| **Predictors** |  | **Estimates** | **CI** | ***P* Value** | **Estimates** | **CI** | ***P* Value** |
|  |  |  |  |  |  |  |  |
| **Allocation (Reference Control)** |  |  |  |  |  |  |  |
|  | Intervention | 0.47 | -1.41 to 2.36 | 0.62 | -0.79 | -3.01 to 1.44 | 0.49 |
| **DASS Anxiety Baseline** |  | 0.75 | 0.61 to 0.90 | **<0.001** | 0.55 | 0.37 to 0.72 | **<0.001** |
| **Length Caregiving Years** |  | -0.11 | -0.27 to 0.05 | 0.17 | -0.22 | -0.41 to -0.04 | **0.02** |
| **Caregiver Age** |  | 0.11 | 0.01 to 0.22 | **0.03** | 0.08 | -0.04 to 0.21 | 0.20 |
| **Caregiver Gender (Reference Female)** |  |  |  |  |  |  |  |
|  | Male | -2.27 | -4.79 to 0.25 | 0.08 | -3.01 | -5.98 to -0.03 | **0.05** |
| **Diagnosis (Reference ALS)** |  |  |  |  |  |  |  |
|  | Muscular Dystrophy | 2.38 | -2.46 to 7.23 | 0.33 | 3.70 | -2.02 to 9.43 | 0.20 |
|  | Myopathy | 3.96 | -1.25 to 9.17 | 0.13 | 4.01 | -2.14 to 10.17 | 0.20 |
|  | Other | 0.82 | -4.12 to 5.75 | 0.74 | 3.40 | -2.43 to 9.23 | 0.25 |
|  | Spinal Muscular Atrophy | 5.21 | 0.11 to 10.31 | **0.05** | 7.07 | 1.04 to 13.09 | **0.02** |
| **HMV (Reference Invasive)** |  |  |  |  |  |  |  |
|  | None | -2.81 | -6.06 to 0.44 | 0.09 | -1.58 | -5.41 to 2.26 | 0.42 |
|  | Non-Invasive | -1.39 | -4.48 to 1.70 | 0.38 | 0.88 | -2.77 to 4.53 | 0.63 |
| **Observations** |  | 100 | | | 100 | | |
| **R^2^ / R^2^ adjusted** |  | 0.578 / 0.525 | | | 0.405 / 0.330 | | |

Abbreviations: ALS, Amyotrophic Lateral Sclerosis; DASS, depression, anxiety and stress scale; HMV, home mechanical ventilation

| **Table 2.** Linear regression models investigating additional predictors of the Depression Anxiety Stress Scale stress measure at 12 and 24 weeks controlling for baseline scores. | | | | | | | |
| --- | --- | --- | --- | --- | --- | --- | --- |
|  |  | **DASS Stress 12 Weeks** | | | **DASS Stress 24 Weeks** | | |
| **Predictors** |  | **Estimates** | **CI** | ***P* Value** | **Estimates** | **CI** | ***P* Value** |
|  |  |  |  |  |  |  |  |
| **Allocation (Reference Control)** |  |  |  |  |  |  |  |
|  | Intervention | 0.01 | -2.16 to 2.18 | 0.99 | -0.78 | -3.28 to 1.72 | 0.54 |
| **DASS Stress Baseline** |  | 0.77 | 0.62 to 0.92 | **<0.001** | 0.62 | 0.45 to 0.79 | **<0.001** |
| **Length Caregiving Years** |  | -0.08 | -0.26 to 0.11 | 0.41 | -0.08 | -0.29 to 0.13 | 0.47 |
| **Caregiver Age** |  | 0.11 | -0.01 to 0.23 | 0.06 | -0.02 | -0.16 to 0.11 | 0.74 |
| **Caregiver Gender (Reference Female)** |  |  |  |  |  |  |  |
|  | Male | -1.56 | -4.44 to 1.33 | 0.29 | -4.82 | -8.15 to -1.49 | **0.005** |
| **Diagnosis (Reference ALS)** |  |  |  |  |  |  |  |
|  | Muscular Dystrophy | 3.56 | -1.99 to 9.10 | 0.21 | -0.89 | -7.28 to 5.50 | 0.78 |
|  | Myopathy | 5.32 | -0.65 to 11.29 | 0.08 | -1.01 | -7.89 to 5.87 | 0.77 |
|  | Other | 1.97 | -3.70 to 7.65 | 0.49 | -1.12 | -7.67 to 5.42 | 0.73 |
|  | Spinal Muscular Atrophy | 5.40 | -0.43 to 11.23 | 0.07 | 3.14 | -3.58 to 9.86 | 0.36 |
| **HMV (Reference Invasive)** |  |  |  |  |  |  |  |
|  | None | -0.42 | -4.14 to 3.31 | 0.82 | -2.32 | -6.61 to 1.98 | 0.29 |
|  | Non-Invasive | -0.89 | -4.43 to 2.65 | 0.62 | 0.11 | -3.97 to 4.19 | 0.96 |
| **Observations** |  | 100 | | | 100 | | |
| **R^2^ / R^2^ adjusted** |  | 0.581 / 0.529 | | | 0.480 / 0.415 | | |

Abbreviations: ALS, Amyotrophic Lateral Sclerosis; DASS, depression, anxiety and stress scale; HMV, home mechanical ventilation

| **Table 3.** Linear regression models investigating additional predictors of the Caregiver stress scale caregiver competence at 12 and 24 weeks controlling for baseline scores. | | | | | | | |
| --- | --- | --- | --- | --- | --- | --- | --- |
|  |  | **CSS Caregiver Competence 12 Weeks** | | | **CSS Caregiver Competence 24 Weeks** | | |
| Predictors |  | Estimates | CI | *P* Value | Estimates | CI | *P* Value |
|  |  |  |  |  |  |  |  |
| Allocation (Reference Control) |  |  |  |  |  |  |  |
|  | Intervention | 0.03 | -0.71 to 0.77 | 0.93 | 0.20 | -0.78 to 1.17 | 0.69 |
| CCS Baseline |  | 0.63 | 0.47 to 0.79 | **<0.001** | 0.46 | 0.25 to 0.68 | **<0.001** |
| Length Caregiving Years |  | 0.01 | -0.05 to 0.07 | 0.74 | 0.07 | -0.01 to 0.15 | 0.11 |
| Caregiver Age |  | -0.01 | -0.05 to 0.03 | 0.56 | -0.04 | -0.09 to 0.01 | 0.13 |
| Caregiver Gender (Reference Female) |  |  |  |  |  |  |  |
|  | Male | -0.10 | -1.06 to 0.86 | 0.83 | -0.57 | -1.84 to 0.71 | 0.38 |
| Diagnosis (Reference ALS) |  |  |  |  |  |  |  |
|  | Muscular Dystrophy | -0.28 | -2.12 to 1.57 | 0.77 | -1.56 | -4.01 to 0.89 | 0.21 |
|  | Myopathy | -1.49 | -3.48 to 0.49 | 0.14 | -2.68 | -5.32 to -0.05 | **0.05** |
|  | Other | 0.11 | -1.77 to 1.98 | 0.91 | -1.37 | -3.86 to 1.12 | 0.28 |
|  | Spinal Muscular Atrophy | -0.29 | -2.24 to 1.66 | 0.77 | -2.04 | -4.63 to 0.55 | 0.12 |
| HMV (Reference Invasive) |  |  |  |  |  |  |  |
|  | None | -1.03 | -2.29 to 0.23 | 0.11 | -1.50 | -3.18 to 0.18 | 0.08 |
|  | Non-Invasive | -0.69 | -1.87 to 0.49 | 0.25 | -0.91 | -2.49 to 0.66 | 0.25 |
| Observations |  | 100 | | | 100 | | |
| R^2^ / R^2^ adjusted |  | 0.502 / 0.439 | | | 0.309 / 0.222 | | |

Abbreviations: ALS, Amyotrophic Lateral Sclerosis; CSS, caregiver stress scale; HMV, home mechanical ventilation
